# Clinical efficacy analysis of high-intensity focused ultrasound ablation in treating fibroadenomas of different breast gland types: a retrospective study

**DOI:** 10.7717/peerj.21025

**Published:** 2026-04-10

**Authors:** Dongmin Yu, Kang He, Zhiqin Lin, Yao Luo, Cai Zhang, Meifang Li

**Affiliations:** 1 First Affiliated Hospital of Gannan Medical University, Ganzhou, China; 2 Chongqing Haifu Hospital, Chongqing, China; 3 State Key Laboratory of Ultrasound in Medicine and Engineering, Chongqing Medical University, Chongqing, China

**Keywords:** High-intensity focused ultrasound ablation, Breast fibroadenoma, Breast gland types, Reduction rate, Clinical efficacy

## Abstract

**Background:**

High-Intensity Focused Ultrasound (HIFU) is an emerging non-invasive therapy for breast fibroadenoma. However, its efficacy across different breast glandular types, which may influence acoustic energy deposition, remains underexplored.

**Objective:**

This study sought to investigate the treatment efficacy of ultrasound (US)-guided HIFU for breast fibroadenoma with respect to different breast gland types.

**Methods:**

A retrospective analysis was conducted on 201 patients (314 lesions) with biopsy-confirmed breast fibroadenomas treated between January 2024 and November 2024. Patients were stratified into four groups based on the American College of Radiology (ACR) breast composition classification: fatty group (<25% glandular tissue), loose group (25–50%), mixed group (51–75%), and dense group (>75%). All patients underwent ultrasound-guided HIFU ablation using the JC-200 system (Chongqing Haifu Medical Technology Co., Ltd., China). The baseline characteristics of patients, lesion features of fibroadenomas, relevant parameters during HIFU treatment, follow-up duration, and tumor volume reduction rate (VRR) were recorded and comparatively analyzed. Statistical analyses included One-Way Analysis of Variance (ANOVA) with least significant difference (LSD)-t tests for pairwise comparisons.

**Results:**

The average surgical power in the loose group (142.8 ± 38.5 W) was significantly higher than that in the fatty group (128.7 ± 23.3 W), mixed group (128.8 ± 36.6 W), and dense group (121.4 ± 39.5 W), with statistically significant differences (*P* < 0.05). The post-HIFU treatment average VRRs for the fatty, loose, mixed, and dense groups were 72.9 ± 16%, 61.6 ± 24%, 55 ± 22%, and 49 ± 32%, respectively. LSD-t test analysis revealed statistically significant differences between pairwise comparisons, including fatty *vs*. loose, fatty *vs*. mixed, fatty *vs*. dense, and loose *vs*. dense groups (*P* < 0.05).

**Conclusion:**

HIFU is a safe and effective treatment for fibroadenomas across different breast gland types. The significantly superior VRR in fatty-type breasts underscores the critical role of preoperative breast gland typing in predicting HIFU outcomes. These findings advocate for the integration of breast density assessment into clinical decision-making to optimize individualized HIFU treatment planning for benign breast tumors.

## Introduction

Fibroadenoma of the breast is the most common benign breast tumor, composed of fibrous and glandular tissues (Weaver & Stuckey, 2022). The development of fibroadenoma is associated with excessive estrogen stimulation. Although most fibroadenomas exhibit slow growth, 0.02–0.125% may undergo malignant transformation (El-Wakeel & Umpleby, 2003). In the early stages, patients often present without obvious symptoms; however, prolonged absence of effective treatment can impair normal work and life activities and impose a significant psychological burden, potentially leading to anxiety and physical discomfort (Kaufman et al., 2002; Li et al., 2018). Surgical excision remains the most effective and definitive treatment for breast fibroadenoma; however, traditional open excision carries risks such as postoperative bleeding, infection, changes in breast contour, and damage to mammary ducts (Yue et al., 2023). Additionally, scars at the surgical site may significantly affect breast aesthetics. Vacuum-assisted minimally invasive rotary excision results in minimal epidermal trauma and reduced intraoperative bleeding but may cause significant internal glandular injury, potentially damaging mammary ducts and affecting breastfeeding. For patients with small breasts or lesions located near the nipple, axilla, or chest wall areas, complete removal may be challenging. Furthermore, this technique is contraindicated in patients with bleeding tendencies or coagulation disorders associated with hematologic diseases (Huo et al., 2016).

High-Intensity Focused Ultrasound (HIFU) is currently the only non-invasive percutaneous ablation technique reported for the treatment of various solid tumors (Izadifar et al., 2020). Compared with minimally invasive thermal ablation modalities such as Radiofrequency Ablation (RFA), Microwave Ablation (MVA), and Laser Ablation (LA), which require interventional ablation probes, HIFU represents a more minimally invasive and precise ablation technology. During HIFU treatment, the transducer generates multiple high-intensity ultrasound beams that penetrate tissues and converge at the target tumor site. This focuses ultrasound energy to a sufficient intensity, generating instantaneous high temperatures at the focal region to induce necrosis of tumor cells within the target area while preserving surrounding normal tissues. Currently, HIFU has been effectively utilized in the treatment of both benign and malignant breast tumors (Liang et al., 2023; Wu et al., 2003). For breast fibroadenomas, HIFU provides several clinical advantages, including no incision or bleeding, preservation of skin integrity, maintenance of breast aesthetics, and conservation of internal structural integrity.

Liang et al. (2023), Yue et al. (2023) have confirmed the feasibility of HIFU ablation for breast fibroadenomas and demonstrated favorable pathological outcomes. In a stratified analysis of fibroadenomas of varying sizes, Wu et al. (2025) also verified the safety and efficacy of HIFU treatment. However, despite this established evidence, a critical gap remains in the literature. The female breast is an organ composed of varying proportions of glandular and adipose tissues, classified by the American College of Radiology (ACR) into distinct density categories (*e.g*., fatty, loose, mixed, and dense) (Spak et al., 2017). Different breast glandular types exhibit variations in tissue density, structural composition, and physiological characteristics. These differences may lead to distinct patterns in the extent of post-ablation tumor necrosis, the risk of local recurrence, and the processes of breast tissue repair and remodeling. To date, comparative analyses specifically investigating the efficacy and safety of HIFU across different breast glandular types remain notably limited.

To address this gap, this study aims to evaluate the efficacy and safety of ultrasound-guided HIFU ablation for fibroadenomas in various types of breast glandular tissue. Additionally, it seeks to investigate clinical outcome differences among these types following HIFU treatment, thereby providing theoretical support and optimizing clinical protocols for focused ultrasound ablation surgery in the management of benign breast tumors.

## Patients and Methods

### Patient characteristics and inclusion criteria

This study was approved by the Ethics Committee of the First Affiliated Hospital of Gannan Medical University (LLSC-2025337), and written informed consent was obtained from all participants. Between January 2024 and November 2024, a total of 201 patients with 314 breast fibroadenomas eligible for HIFU treatment were included. The mean age was 34.4 ± 10.4 years, and the mean body mass index (BMI) was 21.7 ± 3.0 kg/m^2^ (Table 1).

**Table 1:** General information of patients.

Variables	Date
No. of patients (*N*)	201
No. of breast fibroadenomas (*n*)	314
Age (years)	34.4 ± 10.4
BMI (Kg/m^2^)	21.7 ± 3.0

**Inclusion criteria:** (1) Patients aged ≥18 years, (2) Breast ultrasound findings categorized as BI-RADS category 3, (3) Diagnosis of breast fibroadenoma confirmed by core needle biopsy, (4) Lesion diameter ranging from 5 to 40 mm.

**Exclusion criteria:** (1) Pathologically confirmed breast cancer, (2) Pregnant or lactating women, (3) Severe scars in the acoustic pathway of HIFU treatment (scar width > 15 mm and elevated above surrounding skin, with ultrasound showing posterior acoustic shadowing) or metallic markers present following breast cancer radiotherapy.

### Grouping of study subjects

According to the ACR, breast glandular tissue is categorized into four types: ① Fatty type (almost entirely composed of fatty tissue, with glandular tissue accounting for less than 25%), ② Scattered fibroglandular type (with scattered glandular tissue within the breast, comprising 25–50%), ③ Heterogeneously dense type (breast appears unevenly dense, with glandular tissue accounting for 51–75%), and ④ Extremely dense type (breast tissue is highly dense, with glandular tissue exceeding 75%). Based on this classification, enrolled patients were stratified into four groups: fatty group, loose group, mixed group, and dense group.

### Treatment Methods-HIFU

#### Therapeutic system

The treatment was performed using a HIFU system (JC-200 HIFU Ablation System, Chongqing Haifu Medical Technology Co., Ltd., Chongqing, China). The parameters of the focused ultrasound transducer were as follows: Serial Number: H1PA23049CGG; Model: H-type; Frequency: 1 MHz; Focal length: 115 mm; Axial focal dimensions: 6 mm; Focal area (pressure): 1.2 mm × 1.2 mm.

#### Patient preparation

Preoperative localization was conducted using a hand-held mobile ultrasound device, and the skin overlying the breast was marked accordingly. Following local infiltration anesthesia around the breast fibroadenoma, a specialized ultrasound-transparent membrane was applied to the skin to ensure optimal acoustic coupling and to stabilize the breast position. Subsequently, the patient was placed in a prone position on the HIFU treatment bed, with the breast immersed in degassed water. The degassed water served as an acoustic propagation medium to facilitate ultrasound transmission and aided in breast cooling to prevent skin burns.

#### Treatment planning

Real-time onboard ultrasound imaging was utilized to identify and delineate the target fibroadenoma. A virtual ablation plan was formulated based on the three-dimensional measurements of the tumor. The target volume was systematically subdivided into multiple consecutive slices perpendicular to the skin surface, with each slice having a thickness of 2–3 mm to ensure complete coverage.

#### HIFU ablation

The ablation procedure commenced from the deep margin of each slice and progressed towards the superficial margin. The initial acoustic power was set at 100 W. The power was subsequently adjusted in real-time based on the following two key feedback parameters: (1) Tissue Grayscale Change: The operator continuously monitored the ultrasound image for the appearance of a hyperechoic region within the target area, which signifies coagulative necrosis and microbubble formation and served as the key visual endpoint for effective ablation. (2) Patient Feedback: Patients were actively queried regarding any sensation of pain or burning, and the power level was guided accordingly to ensure patient comfort and safety. Treatment at a specific irradiation point was terminated once a stable hyperechoic signal was observed at that point. The procedure continued point-by-point and slice-by-slice until the entire planned fibroadenoma volume was covered by hyperechoic changes.

#### Post-ablation efficacy assessment

Immediately following the procedure, treatment efficacy was assessed using the contrast agent sulfur hexafluoride microbubbles. The post-ablation fibroadenoma was defined as the non-perfused volume (NPV) observed after contrast agent injection and was measured using three-dimensional color Doppler ultrasound. The NPV was calculated using the formula: V = πabc/6. The ablation rate was calculated as (NPV/Fibroadenoma Volume) × 100% (Liang et al., 2023).

#### Post-operative care

After treatment, patients were instructed to intermittently apply ice packs to the treated breast in the observation room for 0.5 to 2 h to alleviate edema and discomfort.

#### Parameter documentation

**Figure 1:**
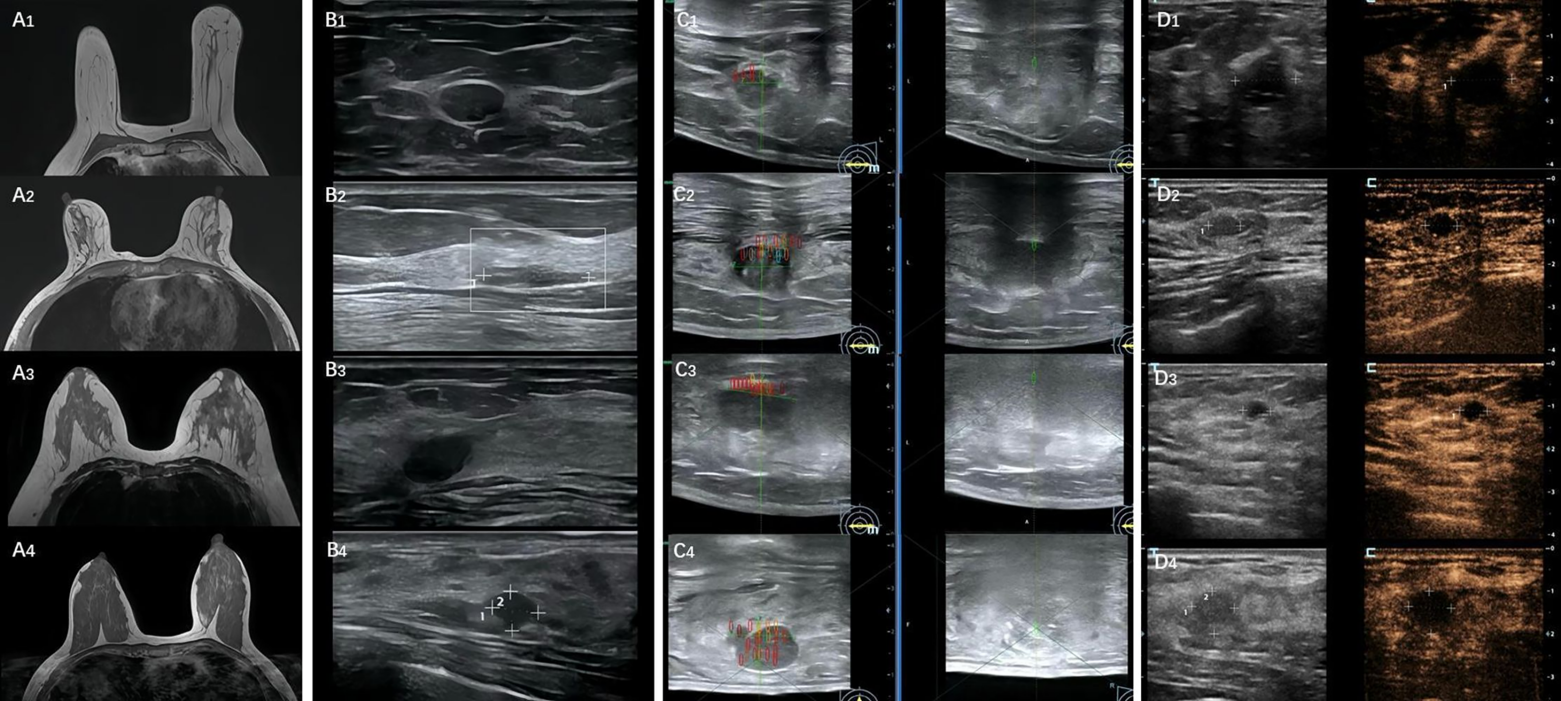
The entire process of HIFU ablation for different breast glandular types. (A1–A4) MRI images of four different breast types; (B1–B4) Ultrasound images of four different breast types; (C1–C4) HIFU intraoperative grayscale variation images for four different breast types (left side: preoperative, right side: immediate postoperative); (D1–D4) Immediate postoperative contrast-enhanced images of HIFU for four different breast types.

Detailed technical parameters for the HIFU treatment of each lesion were recorded, including: localization time (minutes), procedure time (minutes), irradiation time (seconds), average surgical power (W), irradiation ultrasound energy (KJ), Energy Efficiency Factor (EEF, J/mm^3^) calculated as Total Energy/NPV, non-perfused volume (NPV), and ablation rate (%) (see Fig. 1).

### Safety assessment

Detailed records were maintained regarding patient discomfort during and after HIFU treatment. The Visual Analog Scale (VAS) was utilized to evaluate the level of pain experienced during HIFU treatment. Adverse reactions, including pain, skin redness, skin burns, and subcutaneous edema, were closely monitored within 72 h post-HIFU treatment.

### Follow-up and imaging analysis

Follow-up was performed 3 months after treatment, comprising physical examination and ultrasound assessment. Clinical symptoms, including pain, skin pigmentation, nipple discharge, and tumor palpability, were documented. Tumor volume was measured by ultrasound and calculated using the same formula employed in the pre-ablation evaluation.

According to the changes in tumor volume monitored by ultrasound during the follow-up period, the efficacy was classified into four categories: (1) Enlargement: an increase in volume exceeding 10%; (2) Stability: a change in volume within ±10%; (3) Reduction: a decrease in volume exceeding 10%; (4) Complete Disappearance: no detectable lesion on ultrasound examination. The VRR was calculated using the following formula: VRR(%) = [(initial volume-final volume) × 100%]/initial volume.

Tumor volume measurements *via* ultrasound before HIFU ablation, immediately after contrast-enhanced imaging post-procedure, and during follow-up were performed by one primary operator and two senior operators. The average of their measurements was taken to mitigate the influence of human factors on the results. Additionally, the three operators were blinded during data analysis.

## Statistical analysis

Categorical data were presented as frequencies or percentages and compared using the Chi-square test or Fisher’s exact test. Continuous data following a normal distribution were expressed as mean ± standard deviation (X ± SD) and analyzed using One-Way ANOVA, with pairwise comparisons of group means performed using the LSD-t test. The LSD-t test was used for *post-hoc* pairwise comparisons following a significant One-Way ANOVA result, as it provides higher sensitivity for detecting differences when the number of groups is limited and group sizes are unequal. For data with a skewed distribution, the median and interquartile range (P25, P75) were analyzed using the Wilcoxon rank-sum test. All statistical analyses were conducted using SPSS 24.0 software (IBM SPSS, IBM Corporation, Armonk, NY, USA), with *P* < 0.05 considered statistically significant.

## Results

### Comparison of baseline characteristics among patients with different breast types

The adipose group comprised 30 patients with a total of 47 fibroadenomas; the loose group included 33 patients with a total of 49 fibroadenomas; the mixed group consisted of 65 patients with a total of 97 fibroadenomas; and the dense group involved 73 patients with a total of 121 fibroadenomas. Patients in the dense group (mean age: 30.5 ± 10.3 years) were significantly younger than those in the other three groups: adipose group (mean age: 37.6 ± 9.2 years), loose group (mean age: 37.0 ± 10.5 years), and mixed group (mean age: 35.9 ± 10.0 years). Additionally, their body mass index (BMI) was lower, and these differences were statistically significant (*P* < 0.05) (Table 2, Fig. 2).

**Table 2:** Comparison of baseline characteristics of fibroadenoma patients with different breast types.

Variables	Fatty group	Loose group	Mixed group	Dense group	*P*
No. of patients (*N*)	30	33	65	73	–
No. of breast fibroadenomas (*n*)	47	49	97	121	–
Age (years)	37.6 ± 9.2*	37.0 ± 10.5*	35.9 ± 10.0*	30.5 ± 10.3	*P* = 0.001
BMI (Kg/m^2^)	23.9 ± 2.6*^#^	22.7 ± 2.9*	21.8 ± 2.8*	20.4 ± 2.8	*P* < 0.001

**Notes:**

compared with the dense group.

* *P* < 0.05; compared with the mixed group.

# *P* < 0.05.

**Figure 2:**
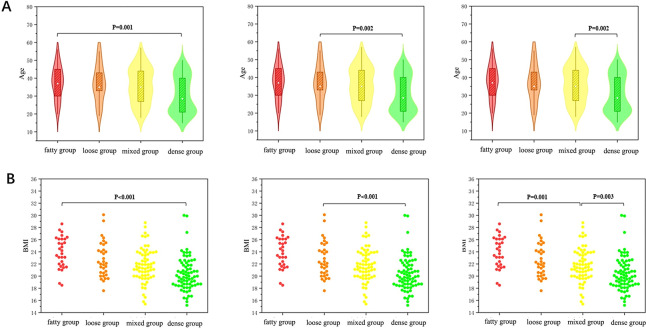
Shows the distribution and differences in patient age and BMI across different breast types. (A) The violin plot illustrates the age distribution and differences among the four patient groups, with the Dense group exhibiting a younger age profile compared to the other groups. (B) The swarm plot demonstrates the distribution and differences in BMI among the four patient groups.

### Characteristics of fibroadenoma lesions

A total of 314 fibroadenomas were identified, with a median size of 10.0 mm (interquartile range: 7.6–13.2 mm) and a median volume of 458.4 mm^3^ (interquartile range: 268.0–1013.1 mm^3^). The mean Distance between the shallow margin of the fibroadenoma and skin was 7.6 ± 5.4 mm, while the median Distance between the deep margin of the fibroadenoma and pectoralis major was 1.2 mm (interquartile range: 0.0–4.5 mm). Adler blood flow classification of ultrasound grading revealed Grade 0 in two cases, Grade I in 221 cases, Grade II in 79 cases, and Grade III in 12 cases (Table 3).

**Table 3:** Baseline characteristics of fibroadenomas in all patients.

Variables	Date
Size of fibroadenomas (mm)	10.0 (7.6, 13.2)
Fibroadenoma volume (mm^3^)	458.4 (268.0, 1,013.1)
Distance between the shallow margin of the fibroadenoma and skin (mm)	7.6 ± 5.4
Distance between the deep margin of the fibroadenoma and pectoralis major (mm)	1.2 (0.0, 4.5)
Adler blood flow classification of ultrasound (0/I/II/III)	2/221/79/12

### Comparison of baseline characteristics of fibroadenomas in different breast types

The size, volume, distance from the superficial margin to the skin, distance from the deep margin to the pectoralis major muscle, and ultrasound Adler blood flow grading of the four groups of fibroadenomas showed no statistically significant differences (Table 4).

**Table 4:** Comparison of baseline characteristics of fibroadenomas in different breast types.

Variables	Fatty group	Loose group	Mixed group	Dense group	P
Size of fibroadenomas (mm)	10.0 (7.9, 14.0)	10.0 (7.5, 12.2)	10.0 (7.2, 12.7)	10.0 (7.6, 14.0)	*P* = 0.475
Fibroadenoma volume (mm^3^)	572.5 (250.0, 1,187.5)	499.5 (292.0, 1,011.8)	457.7 (235.6, 834.6)	433.1 (267.2, 1,138.0)	*P* = 0.703
Distance between the shallow margin of the fibroadenoma and skin (mm)	9.0 ± 3.8	8.1 ± 4.4	7.9 ± 3.1	6.7 ± 7.2	*P* = 0.054
Distance between the deep margin of the fibroadenoma and pectoralis major (mm)	0.0 (0.0, 3.8)	2.6 (0.0, 5.6)	2.0 (0.0, 5.0)	0.0 (0.0, 3.7)	*P* = 0.092
Adler blood flow classification of ultrasound (0/I/II/III)	1/30/16/0	0/29/16/4	0/71/24/2	1/91/23/6	*P* = 0.074

### HIFU treatment results

A total of 314 tumors in 201 patients were successfully treated with HIFU in a single session as planned. The median localization time was 1.0 min (interquartile range: 1.0–2.0 min), and the median procedure time was 16.0 min (interquartile range: 9.0–25.0 min). The average surgical power was 128.1 ± 37.0 W, the median irradiation time was 73.0 s (interquartile range: 47.0–117.0 s), the mean irradiation ultrasound energy was 12.4 ± 14.1 kJ, and the mean energy efficiency factor (EEF) was 24.6 ± 30.8 J/mm^3^. The median non-perfused volume (NPV) percentage reached 83% (interquartile range: 65–104%). For all tumors treated with HIFU, the median visual analog scale (VAS) score was two out of 10 across all patients, with no adverse events such as skin redness, burns, or subcutaneous edema reported (Table 5).

**Table 5:** HIFU treatment outcomes of fibroadenomas in all patients.

Variables	Date
Localization time (min)	1.0 (1.0, 2.0)
Procedure time (min)	16.0 (9.0, 25.0)
Average surgical power (w)	128.1 ± 37.0
Irradiation time (s)	73.0 (47.0, 117.0)
Irradiation ultrasound energy (KJ)	12.4 ± 14.1
EEF (J/mm^3^)	24.6 ± 30.8
NPV (%)	83.3 (65.5, 104.2)
VAS score	2.0 (1.0, 3.0)
Ultrasound energy (*n*)	0

### Comparison of HIFU treatment outcomes for fibroadenomas in different breast types

There were no statistically significant differences among the four groups with respect to HIFU treatment Localization time, procedure time, irradiation time, irradiation ultrasound energy, EEF, NPV, and VAS scores (*P* > 0.05). The average surgical power of the loose group was significantly different compared to the other three groups (Table 6).

**Table 6:** Comparison of HIFU treatment outcomes for fibroadenomas in different breast types.

Variables	Fatty group	Loose group	Mixed group	Dense group	*P*
Localization time (min)	1.0 (1.0, 2.0)	1.0 (1.0, 3.0)	1.0 (1.0, 2.0)	1.0 (1.0, 2.0)	*P* = 0.797
Procedure time (min)	15.0 (7.0, 27.0)	18.0 (11.0, 29.5)	16.0 (9.0, 26.5)	15.0 (8.0, 22.0)	*P* = 0.185
Average surgical power (w)	128.7 ± 23.3	142.8 ± 38.5	128.8 ± 36.6^&^	121.4 ± 39.5^&^	*P* = 0.008
Irradiation time (s)	76.0 (40.0, 155.0)	92.0 (50.5, 147.5)	74.0 (50.5, 118.5)	65.0 (45.5, 101.5)	*P* = 0.103
Irradiation ultrasound energy (KJ)	15.8 ± 26.9	15.2 ± 12.5	11.2 ± 7.2	10.9 ± 11.2	*P* = 0.079
EEF (J/mm^3^)	21.0 ± 17.3	30.2 ± 33.4	29.1 ± 42.1	20.1 ± 21.1	*P* = 0.073
NPV (%)	79.6 (58.3, 98.5)	77.7 (54.2, 97.0)	87.5 (71.1, 117.3)	84.6 (65.8, 103.3)	*P* = 0.088
VAS score	2.0 (2.0, 3.0)	2.0 (2.0, 3.0)	2.0 (1.0,3.0)	2.0 (1.0, 3.0)	*P* = 0.420

**Notes:**

compared with the loose group.

& *P* < 0.05

### Follow-up and volume reduction

Among 201 patients with a total of 314 tumors, the median follow-up duration was 102.0 (93.5, 119.5) days, and the mean volumetric reduction rate (VRR) was 56.7 ± 27.3% (Table 7). Of these tumors, five increased in size, six remained stable, 295 decreased in size, and eight completely resolved.

**Table 7:** Follow-up time and VRR for all patients.

Variables	Date
Follow-up duration (day)	102.0 (93.5, 119.5)
VRR (%)	56.7 ± 27.3

### Comparison of follow-up and volume reduction in fibroadenomas of different breast types

**Figure 3:**
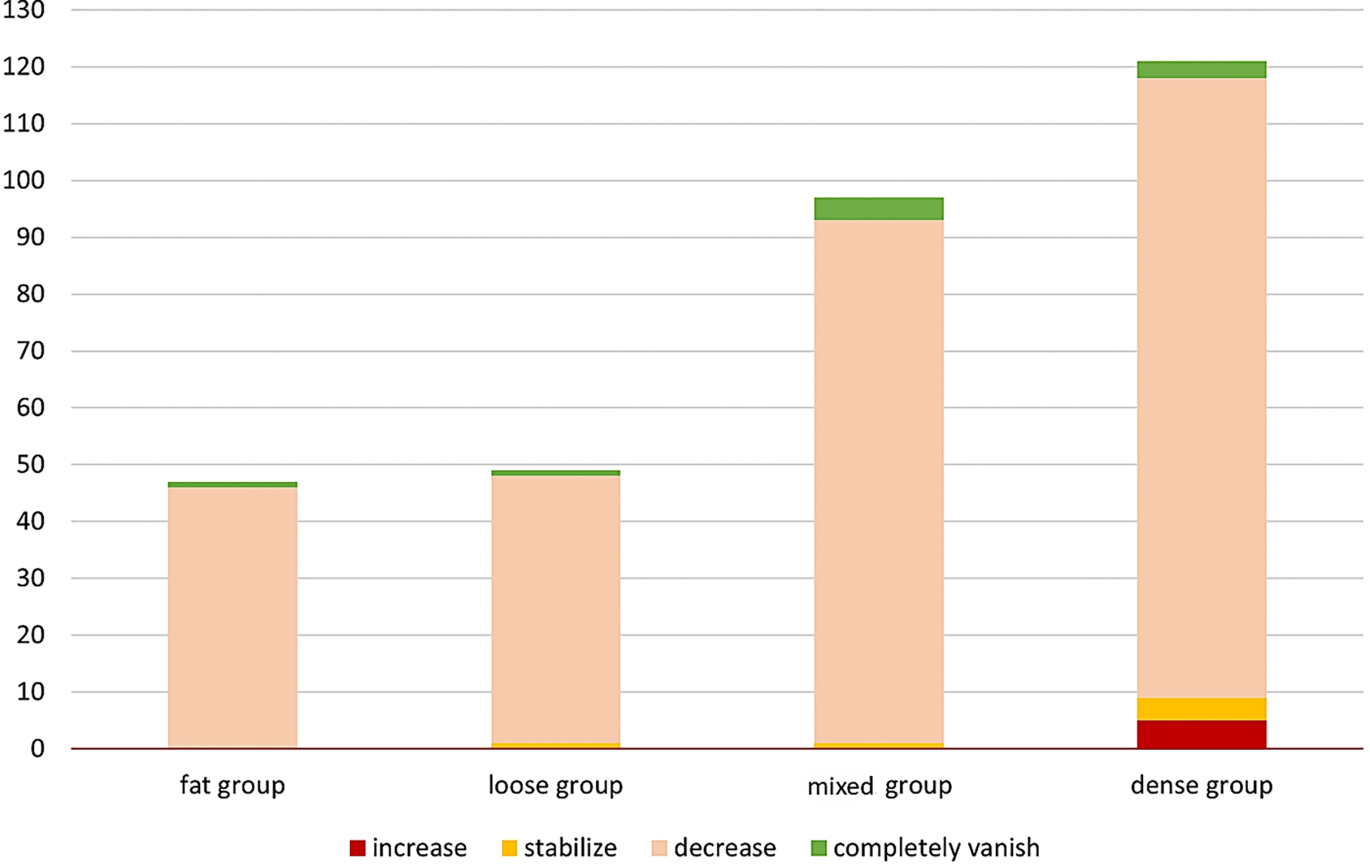
Evaluation of treatment efficacy at 3 months post-HIFU ablation across different breast gland types.

The differences in follow-up times among the four groups were not statistically significant (*P* > 0.05). The mean volumetric reduction rate (VRR) in the fatty group was 72.9 ± 16.0%, 61.6 ± 24.8% in the loose group, 55.4 ± 22.0% in the mixed group, and 49.3 ± 32.2% in the dense group. The differences among the groups were statistically significant (*P* < 0.05) (Table 8). Pairwise comparisons of VRR revealed statistically significant differences between the fatty group and the loose group, the fatty group and the mixed group, the fatty group and the dense group, as well as between the loose group and the dense group (*P* < 0.05) (Table 9). After follow-up, tumor shrinkage was observed in 46 tumors in the fatty tissue type, with one tumor completely disappearing; in the loose tissue type, one tumor remained stable while 47 tumors shrank and one completely disappeared; in the mixed tissue type, one tumor remained stable while 92 tumors shrank and four completely disappeared; in the dense tissue type, five tumors increased in size, four remained stable, and 110 tumors shrank, with three completely disappearing (Fig. 3).

**Table 8:** Comparison of follow-up results after HIFU treatment for different breast types.

Variables	Fatty group	Loose group	Mixed group	Dense group	*P*
Follow-up duration (day)	103.0 (97.0, 135.3)	100.0 (95.5, 110.0)	100.0 (91.5, 114.0)	107.0 (92.5, 127.5)	*P* = 0.166
VRR (%)	72.9 ± 16.0	61.6 ± 24.8 ^†^	55.4 ± 22.0^†^	49.3 ± 32.2^†^^&^	*P* < 0.001

**Notes:**

compared with the fatty group.

† *P* < 0.05;compared with the loose group.

& *P* < 0.05.

**Table 9:** Pairwise comparison of VRR among different breast types using LSD-t test analysis results.

Groups		*P*
Fatty group	Fatty group	*P* = 0.035
	Mixed group	*P* < 0.001
	Dense group	*P* < 0.001
Loose group	Fatty group	*P* = 0.035
	Mixed group	*P* = 0.179
	Dense group	*P* = 0.006
Mixed group	Fatty group	*P* < 0.001
	Loose group	*P* = 0.179
	Dense group	*P* = 0.086
Dense group	Fatty group	*P* < 0.001
	Loose group	*P* = 0.006
	Mixed group	*P* = 0.086

## Discussion

Fibroadenoma of the breast is the most common benign breast tumor in women. Although traditional surgical excision can achieve radical treatment of the lesion, it is associated with drawbacks such as incisional scarring, alteration in breast contour, and a prolonged postoperative recovery period. With advancements in medical technology and evolving treatment paradigms, there is an increasing demand for surgical approaches to shift toward minimally invasive or non-invasive techniques. As an emerging local treatment modality, HIFU has demonstrated distinct advantages in the management of breast diseases. By converting acoustic energy into thermal energy, HIFU induces instantaneous high temperatures at the focal point, resulting in coagulative necrosis of pathological tissues while preserving the integrity of the skin and surrounding structures. Its non-invasive nature not only fulfills patients’ desires for breast aesthetic preservation but also aligns with contemporary surgical trends emphasizing minimally invasive and precise interventions.

This study performed a retrospective analysis of 201 patients (314 fibroadenomas) with a median follow-up duration of 102.0 (93.5, 119.5) days, demonstrating an average VRR of 57.3 ± 27.0%. This indicates superior clinical efficacy compared to the meta-analysis results reported by Wu et al. (2024) (6-month VRR: 52.0%). The discrepancy may be attributed to the individualized treatment protocol utilized in this study, wherein real-time ultrasound imaging was employed to monitor lesion grayscale changes and dynamically adjust HIFU irradiation parameters accordingly. Furthermore, the previous clinical trials included in the aforementioned meta-analysis primarily used the Echopulse treatment system, whereas our center currently utilizes the JC-200 treatment system. Technical differences among various HIFU devices, such as energy output modes, focusing precision, and parameter settings, may account for variations in clinical efficacy and safety.

In the baseline characteristic analysis among patients, those with dense breast tissue exhibited significantly lower age (30.5 ± 10.3 years) and BMI (20.4 ± 2.8 kg/m^2^) compared to other groups (*P* < 0.05). This observation is consistent with the physiological patterns of breast tissue evolution: younger women typically have higher estrogen levels, which promote the proliferation of glandular cells and increase glandular density; individuals with low BMI often have insufficient systemic fat reserves, further contributing to breast tissue densification. Zhang et al. (2022), in their research on the EEF dose prediction model, did not identify direct associations between age or BMI and therapeutic outcomes; similarly, clinical studies on HIFU treatment for abdominal wall endometriosis (Cheng et al., 2024) also support this conclusion. These findings indirectly corroborate our study’s conclusion that age and BMI may not be key determinants of HIFU efficacy.

Although age and BMI did not directly influence HIFU outcomes in this study, their association with breast density suggests that future research should investigate the mechanisms underlying hormone-tissue-treatment response interactions by comprehensively evaluating serum estrogen/progesterone levels and extracellular matrix components within breast tissues. Notably, subgroup analysis revealed a significant impact of breast gland type on treatment efficacy: the VRR at three months for fatty-type breasts (72.9%) was significantly higher than that for dense-type breasts (51.0%), with a statistically significant difference (*P* < 0.05). Despite follow-up periods not yet reaching 6 months, 12 months, or longer, differences in VRR among various breast gland types were already apparent at the three-month observation point. From the perspective of tissue acoustic properties, variations in acoustic impedance among breast types directly affect HIFU energy transmission efficiency. Fatty-type breasts, predominantly composed of adipose tissue (<25% glandular tissue), exhibit lower acoustic impedance and a uniform tissue structure; ultrasound energy experiences minimal attenuation during propagation and focuses more efficiently on the target area, thereby enhancing ablation efficiency (Nasief et al., 2015). In contrast, dense-type breasts (>75% glandular tissue) contain highly compact glandular tissues rich in collagen fibers; sound waves are prone to scattering and energy attenuation during propagation, resulting in uneven HIFU energy deposition and reduced ablation efficacy (D’Astous & Foster, 1986). This disparity in energy attenuation decreases ablation efficiency for dense-type breasts, as reflected by significantly lower VRR values and poorer absorption outcomes. Among participants with stable or enlarged fibroadenomas during follow-up, nearly all cases occurred in the dense group. Furthermore, the VRR for the fatty group (72.9%) was significantly higher than that for the dense group (51.0%), while the loose group (61.6%) and mixed group (55.4%) fell between these two extremes, aligning closely with the gradient changes in breast tissue acoustic properties. Our findings indicate that despite a higher average power level applied to the loose-group patients (142.8 W), their EEF and VRR did not show significant advantages over other groups due to their complex tissue structure (~25–50% glandular composition). This complexity likely caused scattering of focused ultrasound beams and reduced energy deposition efficiency within target areas.

The therapeutic advantages of adipose-type breast tissue may also involve unique tissue repair mechanisms. The immune microenvironment enriched in adipose tissue may synergistically enhance treatment efficacy: adipose-derived stem cells (ADSCs) can differentiate into M2 macrophages (Zhao et al., 2018). Macrophages, as key components of the innate immune system, exhibit two polarized phenotypes: pro-inflammatory M1 and anti-inflammatory reparative M2. Within the adipose tissue microenvironment, ADSCs secrete bioactive substances such as exosomes to induce macrophage differentiation toward the M2 phenotype (Dong et al., 2025). M2 macrophages, also referred to as alternatively activated macrophages, play essential roles in physiological processes including tissue repair, anti-inflammatory responses, and angiogenesis (Shapouri-Moghaddam et al., 2018). When high-intensity focused ultrasound (HIFU) treatment induces coagulative necrosis of fibroadenoma tissues in the breast, damage-associated molecular patterns (DAMPs) are released from the injured area to recruit immune cells to the lesion site (Vénéreau, Ceriotti & Bianchi, 2015). At this stage, M2 macrophages rapidly respond and accumulate extensively at the injury site. On one hand, leveraging their robust phagocytic capacity, M2 macrophages efficiently identify and clear necrotic debris, thereby preventing inflammatory cascades triggered by accumulation and maintaining local microenvironment stability (Arandjelovic & Ravichandran, 2015); on the other hand, they actively secrete various growth factors, such as transforming growth factor-β (TGF-β) and vascular endothelial growth factor (VEGF) (Sica & Mantovani, 2012). TGF-β promotes fibroblast proliferation and collagen synthesis to accelerate fibrous tissue remodeling by constructing a structural framework for tissue repair (Meng, Nikolic-Paterson & Lan, 2016), while VEGF stimulates endothelial cell proliferation and migration, inducing neovascularization that supplies oxygen and nutrients to damaged tissues, significantly accelerating recovery processes (Willenborg et al., 2012).

Furthermore, M2 macrophages secrete anti-inflammatory cytokines, such as interleukin-10 (IL-10), thereby inhibiting the release of pro-inflammatory cytokines, including tumor necrosis factor-alpha (TNF-α) and interleukin-6 (IL-6) (Martinez & Gordon, 2014). This effectively attenuates local inflammatory responses, preventing excessive inflammation from inducing secondary damage to normal tissues and creating optimal conditions for normalization following fibroadenoma ablation. Collectively, these processes contribute to accelerating post-ablation normalization of fibroadenomas, resulting in more pronounced therapeutic outcomes characterized by significant tumor volume reduction and symptom improvement. This study has several limitations. First, the number of patients and lesions differed among the four groups, which may introduce bias into the results. Future studies with larger sample sizes are necessary to validate these subgroup findings. Second, long-term follow-up is required to assess the outcomes of HIFU treatment for different breast types. Fibroadenomas larger than 40 mm were excluded from this study; thus, the efficacy, safety, and prognosis of HIFU treatment for breast fibroadenomas exceeding 40 mm remain unclear. Finally, large-scale multicenter clinical trials are warranted to confirm our findings.

## Conclusion

This study confirms that HIFU is a safe and effective non-invasive approach for treating fibroadenomas across different breast gland types. Most importantly, we identified breast gland type as a significant predictor of HIFU efficacy, with fatty-type breasts demonstrating significantly superior volume reduction compared to dense-type breasts. These findings suggest that preoperative breast imaging assessment is crucial not only for diagnosis but also for predicting HIFU treatment outcomes. Our results provide a foundation for personalized treatment strategies in benign breast disease. Future large-scale, prospective studies with long-term follow-up are warranted to validate these findings and further refine HIFU parameters tailored to specific breast gland types.

## Supplemental Information

10.7717/peerj.21025/supp-1 Supplemental Information 1 Original data of HIFU for different breast types.

## Additional Information and Declarations

### Data Availability

The following information was supplied regarding data availability:

The data is available in the Supplemental File.
